# AOEHO: A New Hybrid Data Replication Method in Fog Computing for IoT Application

**DOI:** 10.3390/s23042189

**Published:** 2023-02-15

**Authors:** Ahmed awad Mohamed, Laith Abualigah, Alhanouf Alburaikan, Hamiden Abd El-Wahed Khalifa

**Affiliations:** 1 Information System Department, Cairo Higher Institute for Languages and Simultaneous Interpretation, and Administrative Science, Cairo 11765, Egypt; 2 Computer Science Department, Prince Hussein Bin Abdullah Faculty for Information Technology, Al Al-Bayt University, Mafraq 25113, Jordan; 3 Hourani Center for Applied Scientific Research, Al-Ahliyya Amman University, Amman 19328, Jordan; 4 Faculty of Information Technology, Middle East University, Amman 11831, Jordan; 5 Applied Science Research Center, Applied Science Private University, Amman 11931, Jordan; 6 School of Computer Sciences, Universiti Sains Malaysia, Pulau Pinang 11800, Malaysia; 7 Department of Mathematics, College of Science and Arts, Qassim University, Al-Badaya 51951, Saudi Arabia; 8 Department of Operations and Management Research, Faculty of Graduate Studies for Statistical Research, Cairo University, Giza 12613, Egypt

**Keywords:** iFogSim, data replication, aquila optimizer, elephant herding optimization, fog computing, IoT, multi-objective optimization

## Abstract

Recently, the concept of the internet of things and its services has emerged with cloud computing. Cloud computing is a modern technology for dealing with big data to perform specified operations. The cloud addresses the problem of selecting and placing iterations across nodes in fog computing. Previous studies focused on original swarm intelligent and mathematical models; thus, we proposed a novel hybrid method based on two modern metaheuristic algorithms. This paper combined the Aquila Optimizer (AO) algorithm with the elephant herding optimization (EHO) for solving dynamic data replication problems in the fog computing environment. In the proposed method, we present a set of objectives that determine data transmission paths, choose the least cost path, reduce network bottlenecks, bandwidth, balance, and speed data transfer rates between nodes in cloud computing. A hybrid method, AOEHO, addresses the optimal and least expensive path, determines the best replication via cloud computing, and determines optimal nodes to select and place data replication near users. Moreover, we developed a multi-objective optimization based on the proposed AOEHO to decrease the bandwidth and enhance load balancing and cloud throughput. The proposed method is evaluated based on data replication using seven criteria. These criteria are data replication access, distance, costs, availability, SBER, popularity, and the Floyd algorithm. The experimental results show the superiority of the proposed AOEHO strategy performance over other algorithms, such as bandwidth, distance, load balancing, data transmission, and least cost path.

## 1. Introduction

Nowadays, cloud computing has become an essential part of the life of companies, large organizations, and big data. The internet of things uses cloud computing to transfer data through sensors in cloud environments [1,2,3,4,5,6,7]. Cloud computing provides many services to users and is pay-to-use. Cloud computing is also used in farms, networks, factories, companies, and other industrial environments [8,9,10,11,12,13,14,15]. The internet of things is also used in data transfer in many large and medium companies, military police, and medicine. Cloud computing consists of infrastructure (IaaS), platform as a service (PaaS), and top-layer software as a service (SaaS) [16,17,18,19,20,21,22]. In addition, cloud computing environments are cheaper than other systems and offer many advantages, such as pay-to-use, scalability, flexibility, high data availability, and system availability. Load balancing across the network is also essential to reduce user waiting time. Cloud computing provides maximum utilization of the available resources and optimal utilization according to each user’s budget [23,24,25,26,27].

Data condensation applications, including static and dynamic replication, relocation from remote geographic locations, and positioning near users were examined. There are three critical problems. (1) What data should be replicated? (2) When should the data be replicated? Finally, (3) where should the new replicas be placed? These are the three main open issues that must be handled for data replication in cloud computing [28]. Determining the optimal and shortest path is vital in addressing moving and positioning replication across nodes near users. Reduce user waiting by balancing data availability across nodes and file high availability across cloud computing. Replication technology saves data, improves performance balance, and fetches files from remote sites according to users’ demands. The proposed strategy also provides data to users from different geographical locations according to their budgets. It also determines the most popular files, and the proposed strategy determines and places them in the path of users [29,30].

### Motivation and Contributions

This work’s motivation is using swarm intelligence to enhance file replication in an IoT-based cloud environment. The main issue of this problem is in the selection and placement data replication process for the least cost path, distance, SBER, reduced time, and cost. This is for quick access to data replication across nodes and the load reduction in the cloud environment. The three problems are: (1) what data should be replicated?; (2) when should the data be replicated?; and (3) where should the new replicas be placed? [28]. These three main open questions must be tackled for data replication in cloud computing. The primary importance is selecting and placing data replication in the data center with less time, response time, and cost, as well as using the least cost path.

The major contributions of the article are given as follows:

- Design a discrete AOEHO strategy for solving the dynamic data replication problem in a fog computing environment.
- Improving a swarm intelligent technique based on the hybrid aquila optimizer (AO) algorithm with the elephant herding optimization (EHO) for solving dynamic data replication problems in the fog computing environment.
- Developing a multi-objective optimization based on the proposed AOEHO to decrease the bandwidth to enhance the load balancing and cloud throughput. It evaluates data replication using seven criteria. These criteria are data replication access, distance, costs, availability, SBER, popularity, and the Floyd algorithm.
- The experimental results show the superiority of the AOEHO strategy performance over other algorithms, such as bandwidth, distance, load balancing, data transmission, and least cost path.

The rest of this paper is organized as follows. Section 2 introduces related work. Section 3 presents the proposed strategy. Section 4 presents the evaluation results—finally, Section 5 presents the conclusion and future work.

## 2. Related Work

Many related studies have researched data replication strategies in the cloud, as follows:

Create a schema for data replication among nodes while preserving privacy and secrecy in fog computing. K. Sarwar et al., in [31] suggested two cross-node replication privacy techniques that were implemented for data security, reliability, and authentication. Compared to other algorithms, the suggested approach fared better regarding memory usage, cost, confidentiality, and privacy.

D. Chen et al., suggested the first decentralized system, BOSSA, which works with all parties on blockchain platforms and shows data retrieval and repeatability. BOSSA also uses privacy-enhancing technology to stop decentralized peers, such as blockchain nodes, from drawing personal conclusions from public data. In order to use intelligent nodes on the Ethereal blockchain, we construct a BOSSA-based prototype and present the security analysis in the context of integrity, privacy, and reliability. Our thorough beta reviews show how workable our suggestion is [32].

The task of time, cost, and energy method optimization plan for task scheduling techniques. C. Li et al., in [33] introduced the Lagrange method to unwinding. This technique considers load balancing, storage, data dependency, data transfer, time, cost, and bandwidth to achieve the shortest data transmission time between nodes. A proposed fault-tolerant task scheduling approach is directed toward cloudlets. The experiments supported the effectiveness of the suggested approach in selecting the best site by the suggested algorithm and transferring data using cloud computing.

T. Shiet et al., presented a novel strategy called multi-cloud application deployment (MCApp). MCApp combines domain-specific big-node search with iterative mixed integer linear programming to streamline the deployment of data replication and user requests. The trials that validated the performance of the suggested approach using actual data and datasets show that MCApp performs noticeably better than other algorithms [34]. A. Majed et al. developed a hybrid strategy for peer-to-peer data replication in cloud environments. It efficiently selected the network’s best and most ideal nodes. Additionally, it chooses and positions the most accessible and often-used user data files. The outcomes of the experiments revealed enhanced network functionality and decreased user waiting [35].

C. LiA et al., suggested an approach based on the Lagrangian relaxation technique for cloud computing’s ideal data replication among nodes. Think about balancing transmission time, bandwidth, and loads. To save money and bandwidth, you can also use the Floyd algorithm. The outcomes demonstrated the suggested algorithm’s superiority to competing algorithms [36].

A. Khelifa et al., introduced a plan for regular and dynamic data replication in cloud computing. The proposed approach intends to decrease the time needed to investigate user requests, achieve load balancing, decrease waiting times, and expedite data access. Additionally, it speeds up data transmission and cloud computing transfer. Additionally, a fuzzy logic approach was implemented for replicating data among nodes using select and placement. It turned out that the suggested algorithm was better than other algorithms [37].

B. Mohammadi et al., presented a cloud computing algorithm for deciding on and configuring data replication among nodes. Reduce user wait times by utilizing the hybrid fuzzy logic and ant colony optimization technique to identify the most appropriate and effective nodes for placement data replication. The suggested algorithm fared better than the competition [38]. An overview of the given studies is presented in Table 1.

## 3. Suggested System and Discussion

### 3.1. Proposed System and Structure

**Figure 1:**
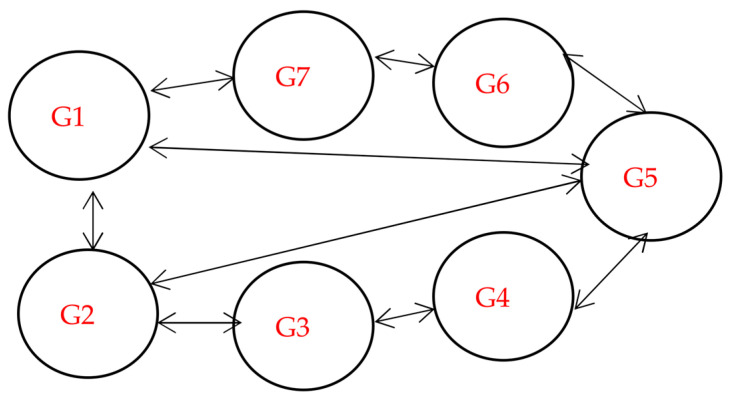
Proposed model for data replication in fog computing.

Figure 1 shows geographically dispersed nodes containing a host, virtual machines (VMS), memory, a CPU, a block, etc. The proposed system comprises file replication, cloudlets, files, blocks, DCs, hosts, VMS, brokers, replica management, and a replica catalog. In order to complete specific activities, such as accessing data replication across nodes or remote geographic locations, the broker acts as a mediator between the user and the DCs. The different DCs, f1, f2, and fn, are filled with many files and randomly dispersed to the other DCs in the following stages. The suggested system is split into two components: choosing and positioning dynamic data replication through the nodes and accessing data using the quickest and least expensive route. To place data replication close to users and select the quickest and the best route to the ideal contract, data replication determination is based on users’ most popular and easily accessible files over time. We combined MOO with HHO to achieve the shortest resource and lowest cost path among nodes in order to maximize cloud computing across nodes.

The data centers can be represented in the model of DCs {dc1, dc2, …, dcs}. The host (PM) can be represented as PMs {pm1, pm2, …, pmy}. The virtual machines can be represented as VMs {vm1, vm2, …, vmz}. File can be represented F= {f1, f2, …, fx}. The block can be represented as B = {b1, b2, …, bm}. Geographically in fog computing, G = {g1, g2, …, gn}.

This section describes the strategy selection and placement of data replication using a hybrid aquila optimizer (AO) algorithm with the elephant herding optimization (EHO) in fog computing. We assume that our proposed system is composed of a certain number of fog nodes (consisting of region point of presences (RPOPs), local point of presences (LPOPs), and gateways), data centers (DCs), internet of things components, and equipment, such as RFID and sensors.

A region point of presence (RPOP) covers different geographical areas in the proposed strategy and a local point of presence (LPOP) in the proposed strategy. Services are deployed on data nodes or proposed fog nodes on IoT sensors. The fog broker, located on the fog nodes layer, is a crucial part of the suggested method. Task manager, resource monitoring service, and task scheduler are the three steps that make up fog broker. Our dynamic data replication approach based on IoT in cloud computing is essential to the fog computing system. A series of configurations are needed to transport data over fog computing, including selecting and placing data replication cross-nodes. We presume that our suggested strategy includes a specific number of fog nodes, such as data centers, IoT services, and DCs. We organized the suggested method from various geographical areas to select and place data replication between nodes in fog computing. Any DCs, fog nodes, or IoT sensors can be used to distribute services. The AO algorithm uses the EHO algorithm to transfer data via DCs with the least cost path and minimum bandwidth. MOO with a Floyd algorithm was also used to reduce the cost, bandwidth, and speed of data transmission in the fog cloud.

### 3.2. Aquila Optimizer (AO)

The aquila, a bird of prey, occupies second place after humans in intelligence because it has a remarkable ability to hunt and has higher capabilities than other animals. The following sections explain the aquila algorithm and how our proposed algorithm works [39].

- Step 1: Expanded exploration

Aquila rises high and detects the area of the place on a large scale, then attacks the prey vertically in the search area. The Equation can be represented as follows:

(1) $$X_{1}\left(t+1\right)=\mathrm{Xbest}\left(t\right)*\left(1-\frac{t}{T}\right)+\left(X_{m}\left(t\right)-\mathrm{Xbest}\left(t\right)*\mathrm{rand}\right)$$

The $X_{m}\left(t\right)$ can also be calculated as follows

(2) $$X_{M}\left(t\right)=\frac{1}{N}\overset{N}{\underset{i=1}{\sum }}X_{i}\left(t\right),\;\forall j=1\ldots \ldots .\mathrm{Dim}$$

Xbest(t) is the best position, $X_{M}\left(t\right)\;\mathrm{is}\;\mathrm{average}\;\mathrm{position}$, t and T are the current iteration and max number of iteration, N is the population size, and R is random between 0 and 1.

- Step 2: Narrowed exploration

Aquila uses short methods to attack the prey within the specified area and circles around the prey. These are the most common ways to obtain and attack prey. The Equation can be represented as follows:

(3) $$X_{2}\left(t+1\right)=\mathrm{Xbest}\left(t\right)*\mathrm{Levy}\left(D\right)+X_{R}\left(t\right)+\left(y-x\right)*\mathrm{rand}$$

(4) $$\mathrm{Levy}\left(D\right)=s*\frac{u*\sigma }{{\left|\nu \right|}^{\frac{1}{\beta }}}\;$$

(5) $$\sigma =\left(\frac{Ӷ\left(1+\beta \right)*\sin e\left(\frac{\pi \beta }{2}\right)}{Ӷ\left(\frac{1+\beta }{2}\right)*\beta *2^{\left(\frac{\beta -1}{2}\right)}}\right)$$

$X_{R}\left(t\right)\;\mathrm{is}\;a\;\mathrm{random}\;\mathrm{position}\;\mathrm{in}\;\mathrm{aquila}$, D is the dimension size, where s and β are constant values equal to 0.01 and 1.5, u and v are random numbers between 0 and 1, and y and x are used to present the spiral shape in the search. It can also be calculated as follows:

(6) y=r*cos(θ)

(7) x=r*sine(θ)

(8) r=r1+U*D1

(9) θ=−w*D1*θ1

(10) $$\theta 1=\frac{3*\pi }{2}$$

where r1 means the number of search cycles between 1 and 20, D1 is composed of integer numbers from 1 to the dimension size (D), and w equals 0.005.

- Step 3: Expanded exploitation

Aquila exploits the selected area of the foraging area and attacks the prey. Aquila uses methods to locate the prey area and attack vertically on it as a primary method. The behavior is represented as follows:

(11) $$X_{3}\left(t+1\right)=\left(\mathrm{Xbest}\left(t\right)-X_{M}\left(t\right)\right)*\;\alpha -\mathrm{rand}+\left(\left(\mathrm{UBj}-\mathrm{LBj}\right)*\mathrm{rand}+\mathrm{LBj}\right)*\delta$$

a and δ are the exploitation adjustment parameters fixed to 0.1 and UBj and LBj are the upper and lower bound of the problem.

- Step 4: Narrowed exploitation

Aquila chases the prey during its escape and the path it takes and attacks it on the ground. The equation can be represented as follows:

(12) $$X_{4}\left(t\right)=\mathrm{QF}*\;\mathrm{Xbest}\left(t\right)-\left(G_{1}*X\left(t\right)*\mathrm{rand}\right)-G_{2}*\mathrm{Levy}\left(D\right)+{\mathrm{rand}*G}_{1}$$

(13) $$\mathrm{QF}\left(t\right)={\;t}^{\left(\frac{2*\mathrm{rand}-1}{{\left(1-T\right)}^{2}}\right)}$$

(14) $$G_{1=2*\mathrm{rand}-1}$$

(15) $$G_{2}=2*\left(1-\frac{t}{T}\right)$$

G1 denotes the movement parameter of aquila is a random number between [–1, 1]. G2 denotes the flight slope when chasing prey. X(t) is the current position, and QF(t) represents the quality function value.

### 3.3. Elephant Herding Optimization

#### 3.3.1. Clan-Updating Operator

Elephants have habits according to their clan, and the mother leads the clan according to their nature [40,41]. The equations can be represented as follows:

(16) $$x_{\mathrm{new},\mathrm{ci},j}=x_{\mathrm{ci},j}+a*\left(x_{\mathrm{best},\mathrm{ci}}-x_{\mathrm{ci},j}\right)*r$$

where x_new_, ci,j and x_ci,j_ present the new and old positions for elephant j in clan ci. x_best,ci_ is matriarch, representing the clan’s best elephant. a is in the range [0, 1], and r is in the range [0, 1]. The best elephant can be represented as follows:

(17) $$x_{\mathrm{new},\mathrm{ci},j}={\;\beta *x}_{\;\mathrm{center},\mathrm{ci}}$$

$x_{\mathrm{center},\mathrm{ci}}$ is the center individual of clan ci, and β is the range [0, 1].

The equations can be represented as follows in the d-th dimension:

(18) $$x_{\mathrm{new},\mathrm{ci},j}=\frac{1}{n_{\mathrm{ci}}}*\;\overset{n_{\mathrm{ci}}}{\underset{j=1}{\sum }}x_{\mathrm{ci},j,d}$$

$x_{\mathrm{ci},j,d\;}$ represents the d-th dimension of the elephant individual, and n_ci_ indicates the number of elephants in clan ci.

#### 3.3.2. Separating Operator

Male elephants leave the family separately when solving problems and improving them. The elephant with the worst fitness of every generation defines a group (class). The behavior can be represented as follows:

(19) $$x_{\mathrm{worst},\mathrm{ci}}=x_{\min}+\left(x_{\max}-x_{\min}+1\right)*\mathrm{rand}$$

where $x_{\max}{\;\mathrm{and}\;x}_{\min}$ upper and lower bound of the individual.${\;x}_{\mathrm{worst},\mathrm{ci}}$ indicates the worst individual in clan ci. Rand is between 0 and 1.

### 3.4. Proposed Swarm Intelligence for Data Replication

This section describes the proposed strategy to define and position replication across nodes in cloud computing environments. For the proposed technique, the shortest path, bandwidth, time, cost, and distance were calculated based on the internet of things via fog computing. Use iFogSim to test the proposed strategy.

#### 3.4.1. Cost and Time of Replication

Cost is a major factor for users to request replication from different geographical locations. The cost varies from one user to another according to the proposed system, the different infrastructure, and the budget of each user. The equation is as follows:

(20) $$\cos t\left({\mathrm{DT}}^{j}\right)=\overset{n}{\underset{y=1}{\sum }}\cos t\left({\mathrm{dt}}_{z}^{y}\right)$$

where

(21) $$\cos t\left({\mathrm{dt}}_{z}^{x}\right)=\overset{m}{\underset{z=1}{\sum }}x_{z}^{y}(p_{z}^{y}+\left(\frac{\mathrm{size}\left({\mathrm{dt}}^{y}\right)}{b_{z}^{y}}\right)*\mathrm{tcost}$$

${\mathrm{DT}}^{i}$ Cost of data set

${\mathrm{dt}}_{z}^{y}$ Data replica in the region

$x_{z}^{y}$ A binary decision variable q ∈(1, 2, 3, ….. l)

$p_{z}^{y}$ Price of replica

$b_{z}^{y}$ Bandwidth network between replicas in the region

#### 3.4.2. Shortest Paths Problem (SPP) between Nodes Based on the Floyd

The problem of choosing and arranging dynamic data replication across a geographically dispersed node to the shortest and best channel in terms of data transmission and bandwidth is addressed in this study [40]. In fog computing, the Floyd finds the shortest path between nodes. The weighted length between the shortest path among the DCs is typically obtained while applying the Floyd algorithm in fog computing. The following is a representation of the equations:

first weighted adjacency matrix A = [ai,j]m × m(22)

ai,j is a path from node I to node j matrix m

The state transition equation is as follows (Equation):

map [I, J]: = min {map [I, k] + map [K, J], map [I, J]}(23)

Map [I, J] demonstrates the shortest distance from I to j.

K is the breakpoint of exhausting I and j.

#### 3.4.3. Popularity Degree of the Data File

Users who access a file frequently, especially recently, determine its popularity. The file that has been located, cloned, and placed between DCs has recently gained much popularity among users. The equation can be shown as follows:

(24) $${\mathrm{PD}}_{i}={\mathrm{an}}_{i}{*w}_{i}$$

Each file’s replication factor (RF_i_) is calculated based on the popularity degree as in Equation (25).

(25) $${\mathrm{RF}}_{i}=\frac{{\mathrm{PD}}_{i}}{{\mathrm{RN}}_{i}{*\mathrm{FS}}_{i}}\;$$

The dynamic threshold (TH) value is calculated as in Equation (23).

(26) $$\mathrm{DH}=\min\left(\left(1-\alpha \right){*\mathrm{RF}}_{\mathrm{system}}\max\left(\begin{array}{c} \forall \\ k\in \left[1,\;2,\;\ldots .,l\right] \end{array}{\;\mathrm{RF}}_{k}\right)\right),\alpha \in \left[0,1\right]$$

${\mathrm{PD}}_{i}$ popularity degree

${\mathrm{an}}_{i}$ number of access

$w_{i}$ time-based forgetting factor

${\mathrm{RF}}_{i}$ replica factor

${\mathrm{RN}}_{i}$ number of replicas

${\mathrm{FS}}_{i}$ size of the data file

#### 3.4.4. System-Level Availability

The system’s overall high availability is known as system byte effective rate (SBER). Tasks for data replication should allow users access to all files. Access to the most popular files is made possible by regular user access. SBER maintains the file’s popularity and accessibility throughout the entire system. An illustration of the equation is as follows:

(27) $$\mathrm{SBER}=\frac{\sum_{i=1}^{s}\left({\mathrm{an}}_{k}\times \left(\sum_{j=1}^{n_{k}}{\mathrm{bs}}_{j})\times \;P\;({\mathrm{FA}}_{k}\right)\right)}{\sum_{i=1}^{s}\left({\mathrm{an}}_{k}\times \left(\sum_{j=1}^{n_{k}}{\mathrm{bs}}_{j}\right)\right)}\;$$

#### 3.4.5. Placement of New Replicas

It places a dynamic data replica between nodes to choose the shortest possible distances. The best minimum path and the least expensive option for consumers are considered while placing data replication across DCs. Additionally, it can be shown as [28,29,30]:

(28) $${\mathrm{br}}_{k}\left({\mathrm{dc}}_{i}\right)=\frac{{\mathrm{RF}}_{k}\left({\mathrm{dc}}_{i}\right)}{\sum_{i=1}^{s}{\mathrm{RF}}_{k}\left({\mathrm{dc}}_{i}\right)}x_{{\mathrm{br}}_{k}\;\left(\mathrm{add}\right)}$$

### 3.5. Computational Complexity

Calculate the time complexity of the proposed strategy AOEHO from tasks IOT application for the number of data repetitions. Calculate the no. of nodes and AO with EHO. Suppose N represents the size of the population, D represents the number of dimensions, T represents the number of tasks, and C represents the cost. The EHO algorithm has a calculated complexity of O(T(D*N + C*N)). Based on the algorithm phase, AOEHO strategy, the time complexity is O(N). Hence, the AOEHO total time complexity is O(N*T*C) and O(N). The main procedure of the proposed method is given in Algorithm 1.
**Algorithm 1: The Proposed Algorithm AOEHO**                  **Input**: Regions, datacenters, data availability, minimum distance between regions, cost, time, SBER, fog nodes, popularity data file, max_iter, population size, and number of IoT tasks.         **Output**: select and place data file replica optimal               **Begin**               Initialize no. of IoT tasks               Initialize the population               Initialize the population using the fitness function               Initialize availability and unavailability probabilities                Initialize replicas according to costs and time               Initialize distance between regions               Initialize popularity data file                Initialize data replication costs and time               Initialize optimal best data replica placement in DC solution               Initialize the least cost path               Initialize SBER               Initialize RF               Initialize budget        **repeat**     **Initialization phase:**     **Initialize** the population X of the AO.     **Initialize** the parameters of the AO.             **WHILE** (t < T)        **Calculate** the fitness function values.     **Determine** the best-obtained solution according to the fitness values (Xbest(t)).              **FOR** (i = 1,2,...,N)     **Update** the mean value of the current solution XM(t).     **Update** the x, y, G1, G2, Levy(D), etc.              **IF** (t ≤ (2/3)*T)              **IF** (rand ≤ 0.5)     **Update** the current solution using Equation (1).                        Step 1: Expanded exploration (X1)              **IF** (Fitness X1(t + 1) < Fitness X(t))                 X(t) = X1(t + 1)                 **IF** (Fitness X1(t + 1) <Fitness (Xbest(t))                   Xbest(t) = X1(t + 1)                           **ENDIF**                           **ENDIF**                                 **ELSE**
       **Update** the current solution using Equation (3).                           Step 2: Narrowed exploration (X2)                  **IF** (Fitness X2(t + 1) <Fitness X(t))                      X(t) = X1(t + 1)                  **IF** (Fitness X2(t + 1) <Fitness (Xbest(t))                      Xbest(t) = X2(t + 1)                             **ENDIF**                             **ENDIF**                             **ENDIF**
                                    **ELSE**
                   **IF** (rand ≤ 0.5)       **Update** the current solution using Equation (11).                                 Step 3: Expanded exploitation (X3)                  **IF** (Fitness X3(t + 1) <Fitness X(t))                       X(t) = X3(t + 1)                   **IF** (Fitness X3(t + 1) <Fitness (Xbest(t))                        Xbest(t) = X3(t + 1)                             **ENDIF**                             **ENDIF**                                     **ELSE**
        **Update** the current solution using Equation (12).                                    Step 4: Narrowed exploitation (X4)                   **IF** (Fitness X4(t + 1) <Fitness X(t))                         X(t) = X4(t + 1)                   **IF** (Fitness X4(t + 1) <Fitness (Xbest(t))                         Xbest(t) = X4(t + 1)                               **ENDIF**                               **ENDIF**                               **ENDIF**                               **ENDIF**                      **ENDFOR**                      **ENDWHILE**                   **Return** the best solution (Xbest).            Apply Elephant Herding Optimization (EHO) to AO   t++                       End while           Calculate the RF           Calculate the distance between regions           Calculate SBER           Calculate the cost and time  **Return** the optimal minimum data replica placement in the region.

## 4. Experimental Evaluation

### 4.1. Configuration Details

The proposed system has been implemented on iFogSim. AOEHO selects and coordinates placement dynamic data replication between fog nodes. In this section, we discuss the configuration and fog cloud for the proposed system. The parameters settings are given in Table 2 [42,43,44].

### 4.2. Results and Discussion

#### 4.2.1. Different Scenarios of Data Replica Size

We created different scenarios of experiments on select and placement data replication, optimal user access, reduced waiting time, and reduced bandwidth. The proposed strategy was compared with three other strategies (MCS, FFRPP, and NSGAII-DRP) to evaluate their effectiveness of the proposed strategy. The proposed strategy is dynamic on IoT devices according to user access requests and choosing the best and most appropriate way to define and place replication over the IoT on cloud computing.

##### First Scenario of Tasks

**Figure 2:**
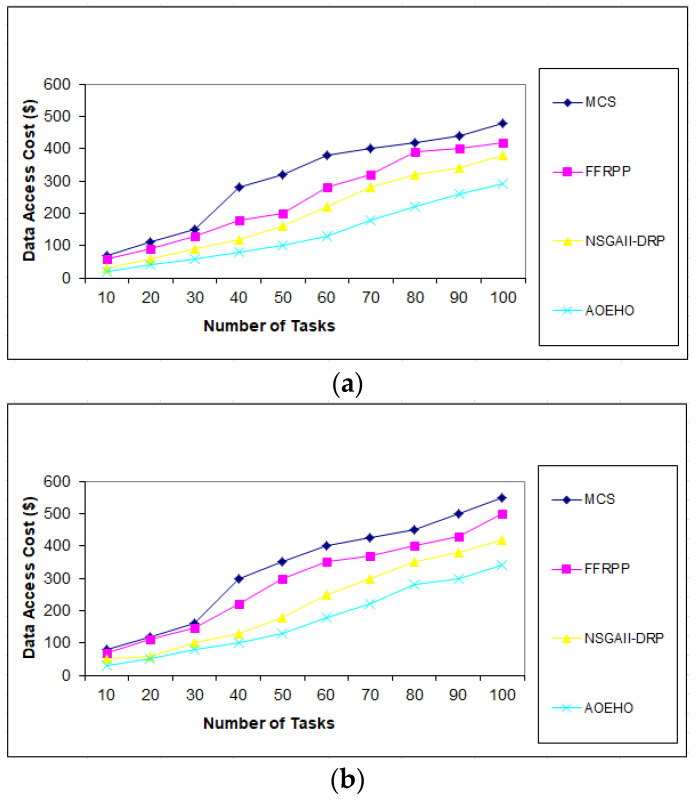
Cost number of tasks and cost = 100 tasks. (**a**) Size of data replication = 64 MB. (**b**) Size of data replication = 320 MB.

**Figure 3:**
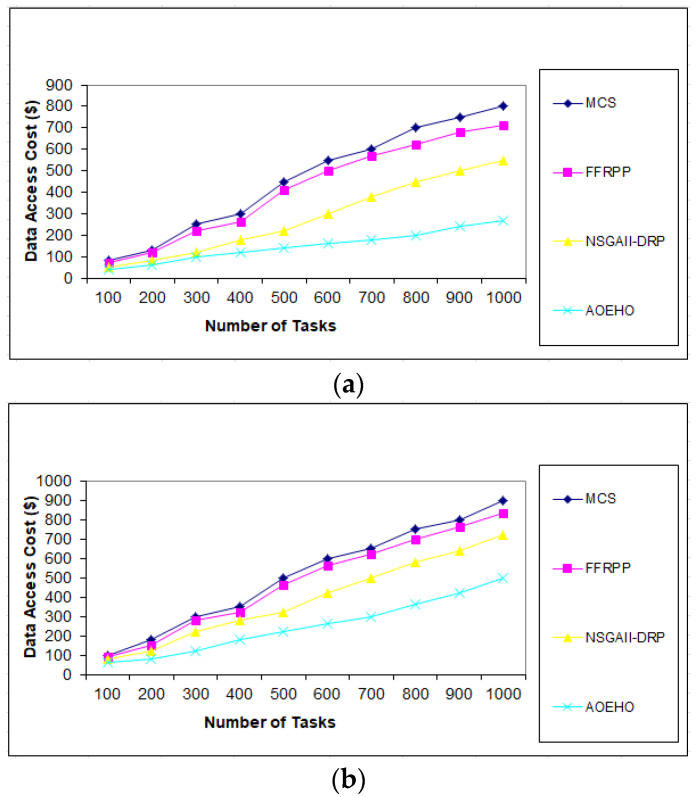
Cost number of tasks and cost = 1000 tasks. (**a**) Size of data replication = 64 MB. (**b**) Size of data replication = 320 MB.

**Figure 4:**
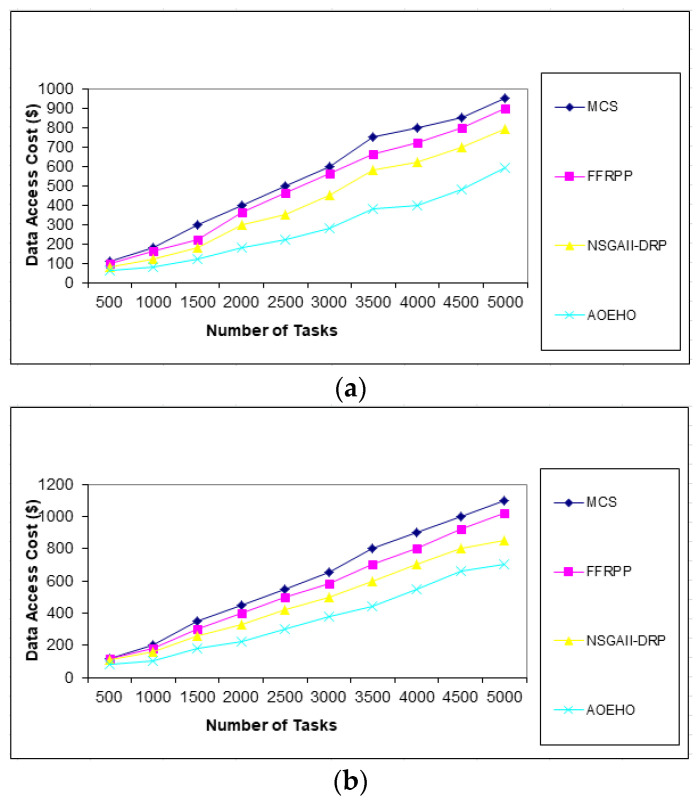
Cost number of tasks and cost = 5000 tasks. (**a**) Size of data replication = 64 MB. (**b**) Size of data replication = 320 MB.

Users submit a set of tasks to optimize data replication under different scenarios. The first scenario contains Figure 2, Figure 3 and Figure 4 of different data replication sizes, such as 64 and 320 MB. The tasks contain a different number according to the proposed strategy, ranging from 10 to 5000, to calculate the access cost for each task according to the proposed strategy. It considers the availability of data and the access time of each replica, determines the version that enjoys high popularity, selects it, and places it in the path of users. The proposed strategy outperformed other strategies in terms of cost deposited by users.

##### Second Scenario of Response Time for Tasks

**Figure 5:**
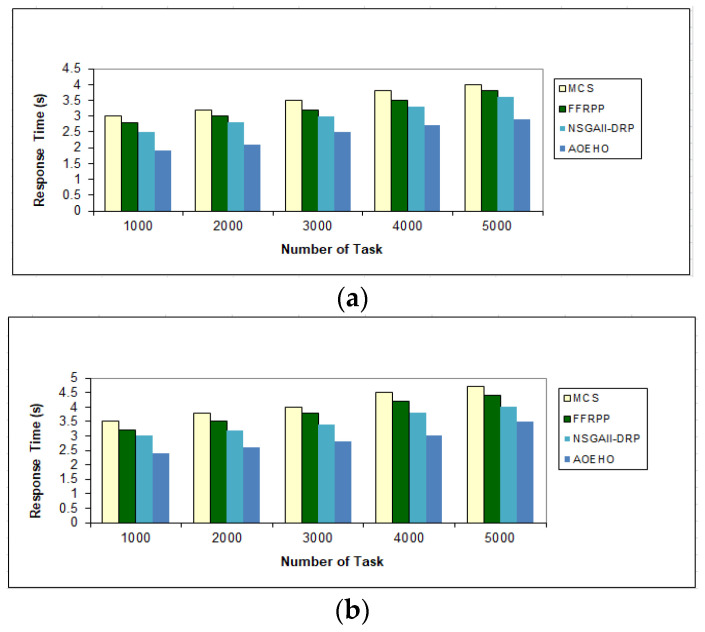
Response time of tasks = 5000 tasks. (**a**) Size of data replication = 64 MB. (**b**) Size of data replication = 320 MB.

Figure 5 shows the data access time with tasks ranging from 1000 to 5000 tasks and selecting files of 64 or 320 MB. Placing files from remote geographical locations and close to users reduces waiting time and speeds up access to optimal replication. The proposed strategy outperformed other strategies in reducing waiting time for users.

##### Third Scenario of Response Time for File

**Figure 6:**
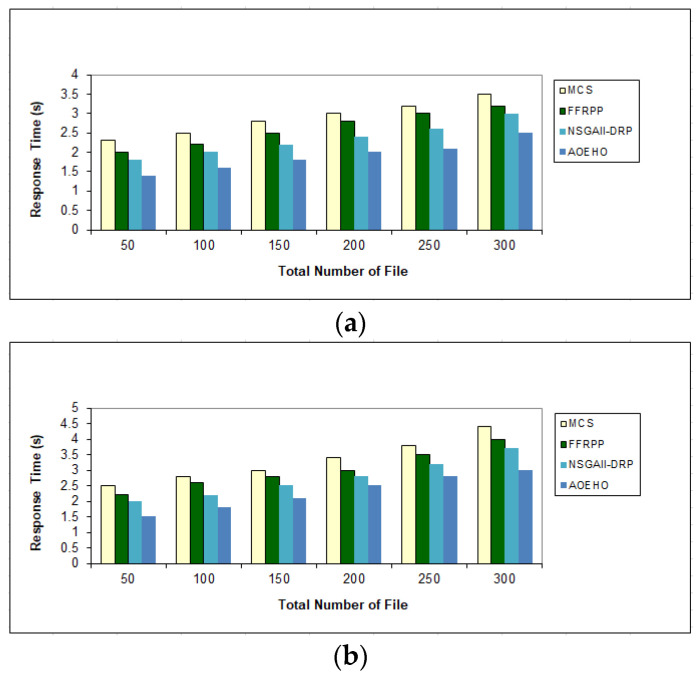
Response time of data file. (**a**) Size of data replication = 320 MB. (**b**) Size of data replication = 320 MB.

Figure 6 shows the number of replications, identifying them and placing them in the path of the two users with the least time to achieve optimal and frequent access to these files from different geographical locations. The optimal nodes will be determined according to the proposed strategy, and the most popular files will be placed to reduce users’ attention time. The proposed strategy outperformed other strategies in reducing waiting time for users.

##### Third Scenario of Execution Time

**Figure 7:**
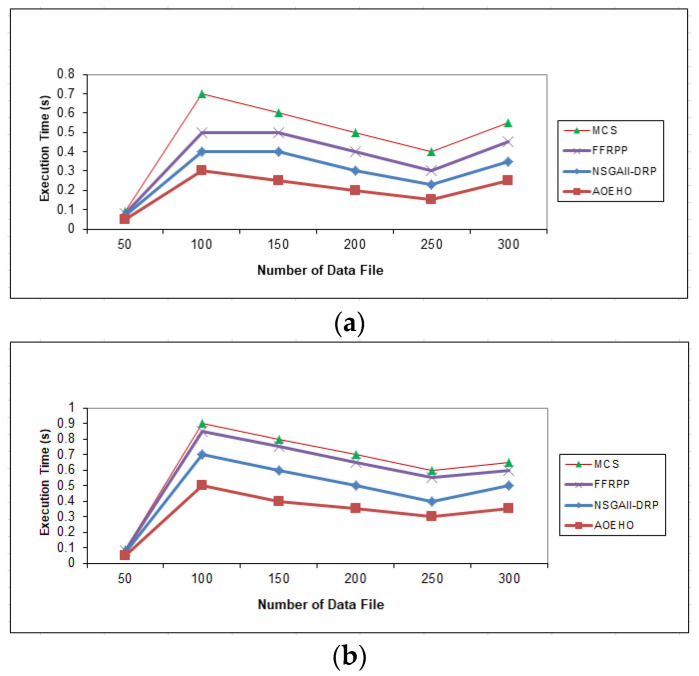
Execution time of data file. (**a**) Size of data replication = 64 MB. (**b**) Size of data replication = 320 MB.

Figure 7 contains the speed implementation of accessing, selecting data replication, and placement of the most popular files across nodes in cloud computing. A scenario of 64 and 320 MB was generated in select and placement data replication to obtain and place optimal replication across cloud computing nodes. The proposed strategy outperformed other strategies in reducing waiting time for users.

##### Fourth Scenario of Data Transmission in Nodes

**Figure 8:**
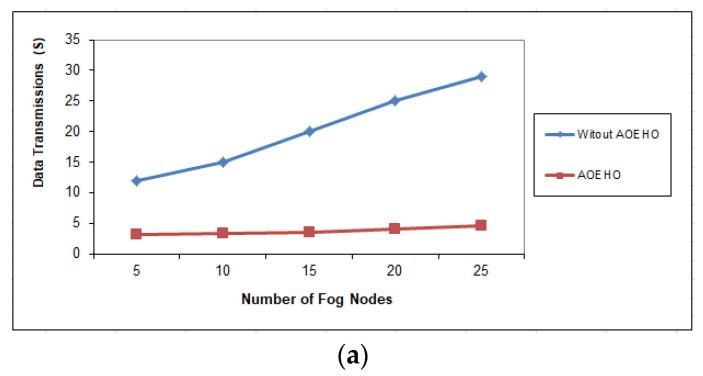

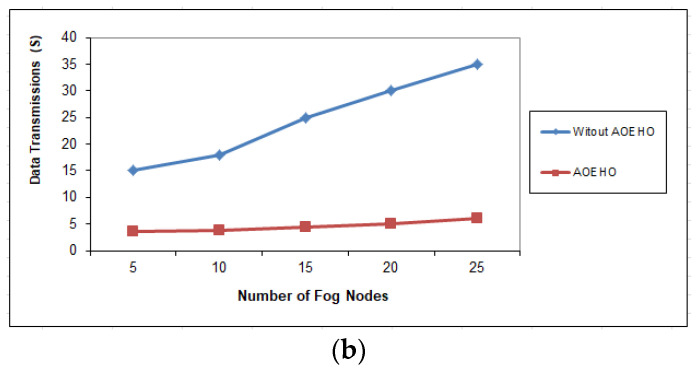
Data transmission between fog nodes = 25 nodes. (**a**) Size of data replication = 64 MB. (**b**) Size of data replication = 320 MB.

**Figure 9:**
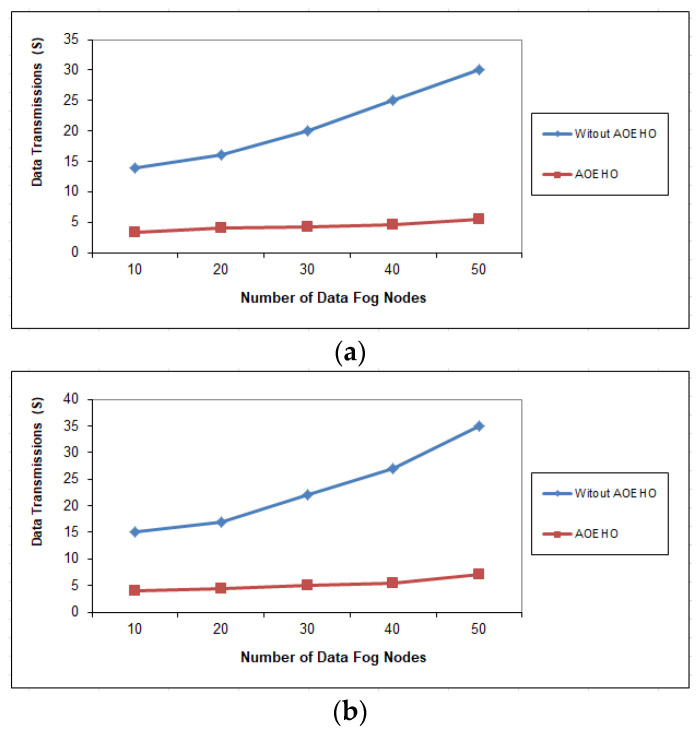
Data transmission between fog nodes = 50 nodes. (**a**) Size of data replication = 64 MB. (**b**) Size of data replication = 320 MB.

**Figure 10:**
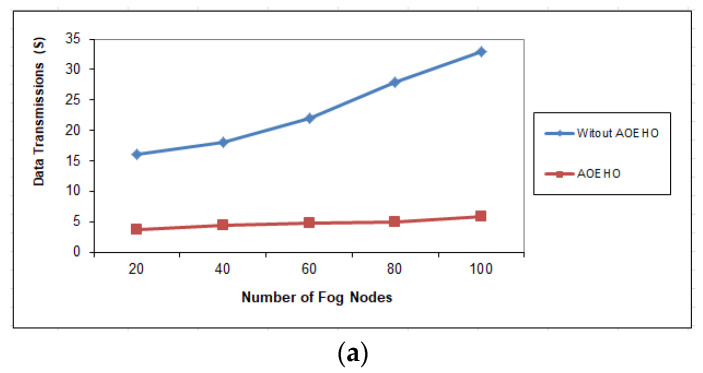

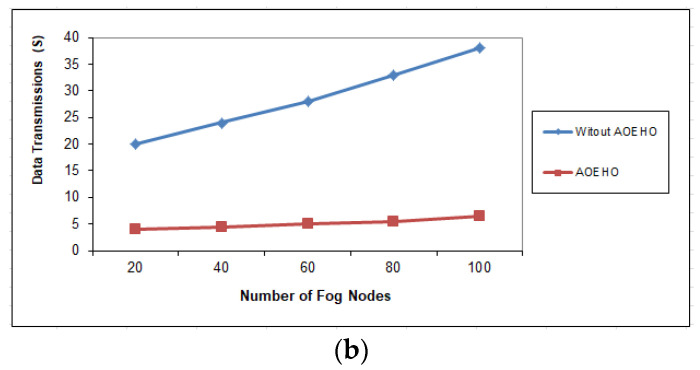
Data transmission between fog nodes = 100 nodes. (**a**) Size of data replication = 64 MB. (**b**) Size of data replication = 320 MB.

Figure 8, Figure 9 and Figure 10 assess the impact of a different number of IoT-based nodes on cloud computing. We considered the number of replication and moved it across nodes in cloud computing with the lowest path and cost. We conducted the process of transferring data from x to 100 nodes and the effect of transferring data across nodes in cloud computing. From the reality of the proposed strategy, the greater the number of nodes, the more significant the improvement of the proposed strategy and the prediction of different ways to achieve the lowest path and cost. The proposed strategy outperformed other strategies in reducing waiting time for users.

##### Fifth Scenario of Data Transmission in Tasks

**Figure 11:**
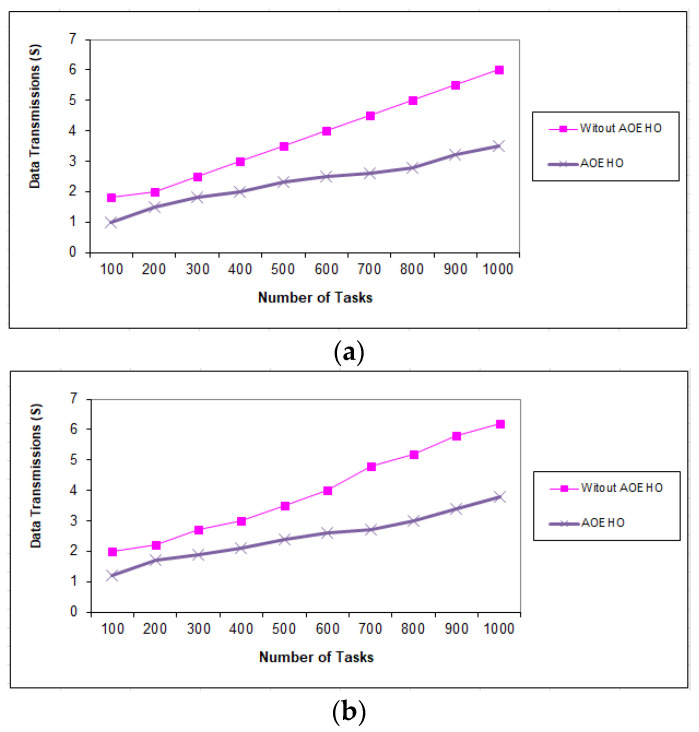
Data transmission between tasks = 1000 tasks. (**a**) Size of data replication = 64 MB. (**b**) Size of data replication = 320 MB.

**Figure 12:**
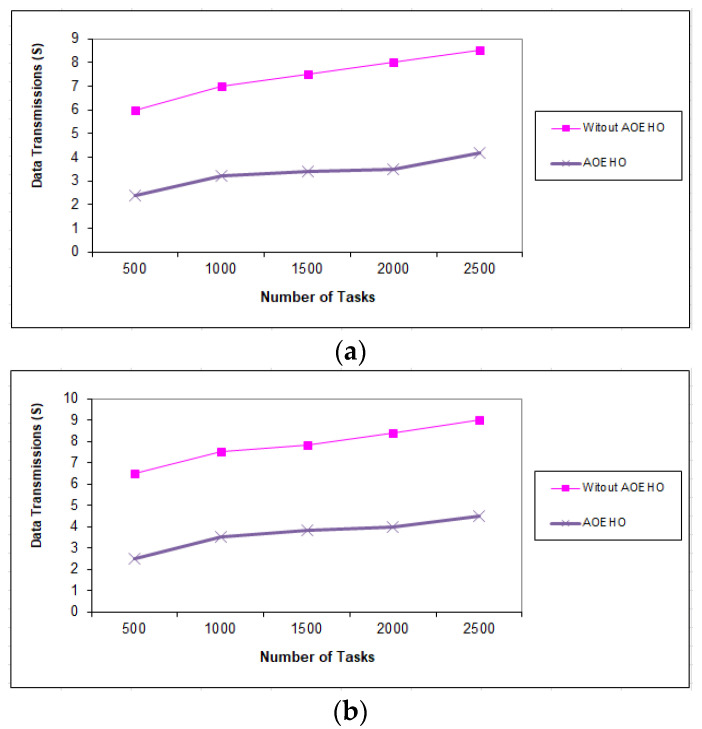
Data transmission between tasks = 2500 tasks. (**a**) Size of data replication = 64 MB. (**b**) Size of data replication = 320 MB.

**Figure 13:**
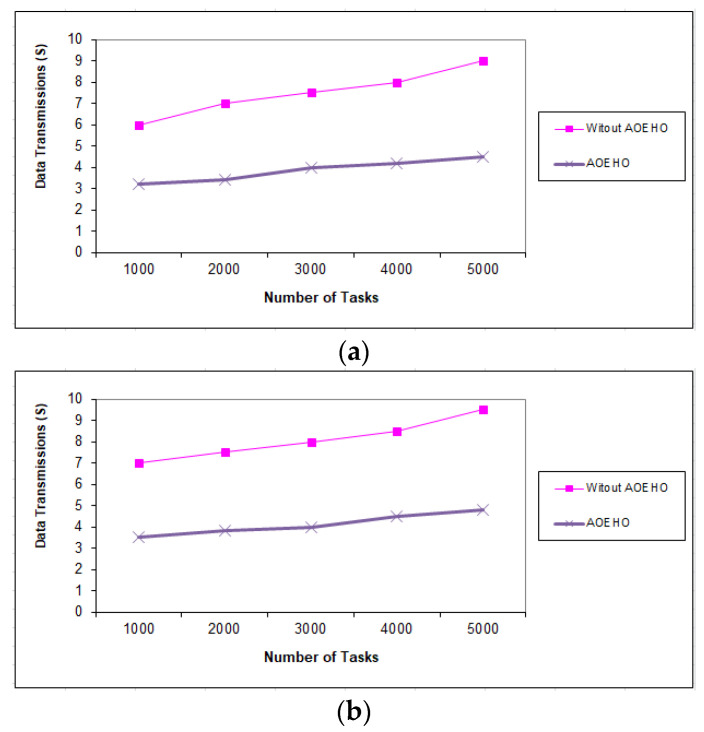
Data transmission between tasks = 5000 tasks. (**a**) Size of data replication = 64 MB. (**b**) Size of data replication = 320 MB.

Figure 11, Figure 12 and Figure 13 evaluate the impact of a different number of IoT-based tasks on cloud computing. We considered the number of replications with sizes of 64 and 320 MB and transferred them across nodes in cloud computing with the lowest path and lowest cost. We conducted data migration from 10 to 5000 tasks and the effect of data migration across nodes in cloud computing. Additionally, the number of files is determined from geographical locations according to the frequent access of users to files. The most popular files are identified by the proposed strategy and placed across the nodes near the users. From the reality of the proposed strategy, the greater the number of tasks, the more significant the improvement of the proposed strategy and the prediction of different ways to achieve the least path and cost. The proposed strategy outperformed other strategies in reducing waiting time for users.

### 4.3. Performance Evaluation

#### 4.3.1. Degree of Balancing

**Figure 14:**
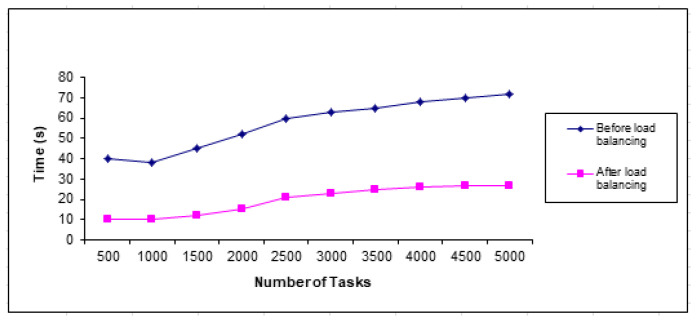
Degree of imbalance.

Figure 14 shows the imbalance over the network fog nodes to perform several different tasks simultaneously. The proposed model lowers the degree of imbalance to a minimum level. The proposed strategy outperformed other strategies in reducing the degree of balancing.

#### 4.3.2. Data Loss Rate

**Figure 15:**
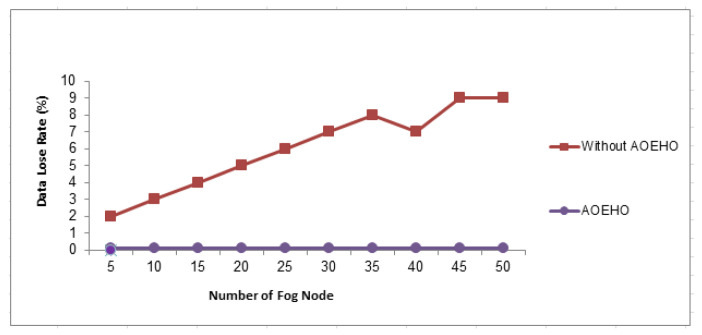
Data lose rate.

Figure 15 shows the data loss rate across nodes in cloud computing. The data transfer rate across 50 nodes reaches a loss rate of 0 in the proposed system. The proposed strategy outperformed other strategies in reducing the data loss rate.

#### 4.3.3. Load Balancing

**Figure 16:**
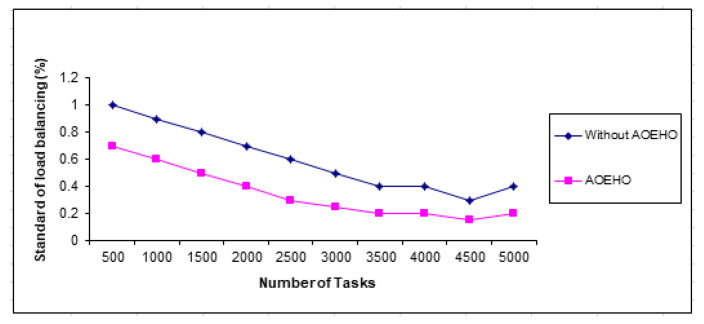
Standard of load balancing.

Figure 16 shows load balancing in different geographic locations decreases with the size of different files in nodes across cloud computing. Five thousand tasks were performed on a data set of different sizes to evaluate the efficiency of load balancing across nodes and the capacity and width of different files across nodes with bandwidth reduction. The proposed strategy outperformed other strategies in reducing load balancing and bandwidth.

#### 4.3.4. Throughput Time

**Figure 17:**
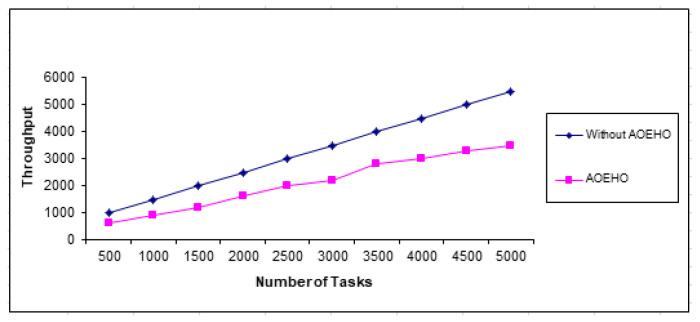
Throughput time for tasks.

The proposed strategy works on adequate access to data, optimal use of resources, and improved productivity. It places data across nodes in cloud computing and the data transfer rate across the proposed system. It achieves significant resource utilization and time and cost savings across nodes in cloud computing. An improvement can also be made to reduce congestion across the network in cloud computing. The proposed strategy outperformed other strategies in reducing load balancing and throughput as shown in Figure 17.

## 5. Conclusions and Future Work

Cloud computing deals with the internet of things to move data, achieve availability, make data available, and improve data access. In this research, we created a hybrid method called the AOEHO strategy to address the optimal and least expensive path and to determine the best replication via cloud computing. The aquila optimizer (AO) algorithm was combined with the elephant herding optimization (EHO) for solving dynamic data replication problems in the fog computing environment. Additionally, a set of objectives was used to improve the balance between nodes and costs across cloud computing. At the same time, AOEHO’s proposed strategy is to find the most popular files and choose the best location for the nodes closest to the users. The proposed strategy also reduces user response time and waiting time. Floyd’s algorithm optimized the shortest and most optimal path to select and place replication across nodes in cloud computing. The proposed AOEHO strategy is superior to other strategies regarding bandwidth, distance, load balancing, data transmission, and the least cost path. The proposed algorithm was simulated and evaluated via iFogSim. In future work, according to the efficiency of the AOEHO strategy, it can be applied to address more optimization problems in real-world implementation, including tasks in data replication, data transmission, routing data replication problems, healthcare, energy optimization problems, and multi-hop data between nodes.

## Figures and Tables

**Table 1:** Comparison of the reviewed select and placement data replication in the cloud environment.

Author	Advantage	Disadvantage
K. Sarwar et al., in [31]	Privacy	High latency
	Secrecy	High bandwidth
	Reliability	load balancing
	Authentication	
D. Chenet al., in [32]	Reliability	High replication cost
	Secrecy	High response time
	Privacy	
C. Li et al., in [33]	load balancing	High response time
	storage	High cost
	data transmission time	
T. Shiet et al., in [34]	High availability	High cost
	High performance	High response time
A. Majed et al., in [35]	decreased user waiting	High cost
	load balancing	High storage cost
	data transmission time	
C. LiA et al., in [36]	data transmission time	High response time
	load balancing	
	low cost	
A. Khelifa et al., in [37]	Low response time	High cost
	data transmission time	High storage cost
	load balancing	
B. Mohammadi et al., in [38]	Low response time	High cost
	load balancing	High storage cost

**Table 2:** Parameters data sets of the system.

Cloud Entity	Ranges
Nodes	[1, 120]
User	[10, 1000]
Regions	[5, 50]
Geographical	[10, 64]
Bandwidth	[2 Mbps, 256 Mbps]
Data sets	[0.1, 64 G]
Data file	[10, 1000]
Cost of file	[100, 5000]
Storage nodes	[8, 512]
Transfer rate	[16, 256 MB/s]
Host	[10, 300]
Processor	[12, 128]
MIPS	[100, 20,000]
Memory RAM	[2, 64 G]
Virtual machine	[100, 1000]
Processor	[8, 128]
MIPS	[200, 20,000]
Memory RAM	[2, 64 G]
Cloudlet	[1000, 6000]
Length of task	[1000, 10,000 MI]

## Data Availability

Data is available from the authors upon reasonable request.
